# Mental Health Chatbot for Young Adults With Depressive Symptoms During the COVID-19 Pandemic: Single-Blind, Three-Arm Randomized Controlled Trial

**DOI:** 10.2196/40719

**Published:** 2022-11-21

**Authors:** Yuhao He, Li Yang, Xiaokun Zhu, Bin Wu, Shuo Zhang, Chunlian Qian, Tian Tian

**Affiliations:** 1 Institute of Applied Psychology College of Education Tianjin University Tianjin China; 2 Laboratory of Suicidology Tianjin Municipal Education Commission Tianjin China; 3 Tianjin Vocational Institute Tianjin China; 4 Tianjin Quesoar Intelligent Technology Co, Ltd Tianjin China; 5 College of Intelligence and Computing Tianjin University Tianjin China

**Keywords:** chatbot, conversational agent, depression, mental health, mHealth, digital medicine, randomized controlled trial, evaluation, cognitive behavioral therapy, young adult, youth, health service, mobile health, COVID-19

## Abstract

**Background:**

Depression has a high prevalence among young adults, especially during the COVID-19 pandemic. However, mental health services remain scarce and underutilized worldwide. Mental health chatbots are a novel digital technology to provide fully automated interventions for depressive symptoms.

**Objective:**

The purpose of this study was to test the clinical effectiveness and nonclinical performance of a cognitive behavioral therapy (CBT)–based mental health chatbot (XiaoE) for young adults with depressive symptoms during the COVID-19 pandemic.

**Methods:**

In a single-blind, 3-arm randomized controlled trial, participants manifesting depressive symptoms recruited from a Chinese university were randomly assigned to a mental health chatbot (XiaoE; n=49), an e-book (n=49), or a general chatbot (Xiaoai; n=50) group in a ratio of 1:1:1. Participants received a 1-week intervention. The primary outcome was the reduction of depressive symptoms according to the 9-item Patient Health Questionnaire (PHQ-9) at 1 week later (T1) and 1 month later (T2). Both intention-to-treat and per-protocol analyses were conducted under analysis of covariance models adjusting for baseline data. Controlled multiple imputation and δ-based sensitivity analysis were performed for missing data. The secondary outcomes were the level of working alliance measured using the Working Alliance Questionnaire (WAQ), usability measured using the Usability Metric for User Experience-LITE (UMUX-LITE), and acceptability measured using the Acceptability Scale (AS).

**Results:**

Participants were on average 18.78 years old, and 37.2% (55/148) were female. The mean baseline PHQ-9 score was 10.02 (SD 3.18; range 2-19). Intention-to-treat analysis revealed lower PHQ-9 scores among participants in the XiaoE group compared with participants in the e-book group and Xiaoai group at both T1 (*F*_2,136_=17.011; *P*<.001; *d*=0.51) and T2 (*F*_2,136_=5.477; *P*=.005; *d*=0.31). Better working alliance (WAQ; *F*_2,145_=3.407; *P*=.04) and acceptability (AS; *F*_2,145_=4.322; *P*=.02) were discovered with XiaoE, while no significant difference among arms was found for usability (UMUX-LITE; *F*_2,145_=0.968; *P*=.38).

**Conclusions:**

A CBT-based chatbot is a feasible and engaging digital therapeutic approach that allows easy accessibility and self-guided mental health assistance for young adults with depressive symptoms. A systematic evaluation of nonclinical metrics for a mental health chatbot has been established in this study. In the future, focus on both clinical outcomes and nonclinical metrics is necessary to explore the mechanism by which mental health chatbots work on patients. Further evidence is required to confirm the long-term effectiveness of the mental health chatbot via trails replicated with a longer dose, as well as exploration of its stronger efficacy in comparison with other active controls.

**Trial Registration:**

Chinese Clinical Trial Registry ChiCTR2100052532; http://www.chictr.org.cn/showproj.aspx?proj=135744

## Introduction

The COVID-19 pandemic has had a huge impact on people’s mental health, increasing the rates of depression and anxiety by more than 25% globally in the first year, with people aged 20-24 years being more affected than older people [1]. However, there are still many limitations in traditional face-to-face psychotherapy and mental health services, including expensive treatment, geographical limitations, few experienced therapists, and delayed treatment [2], and stigma is considered as the most significant barrier to providing mental health services [3,4]. Limited accessibility and acceptability were more obvious with the rising risk of mental health problems [5,6] led by quarantine and social isolation during the COVID-19 pandemic [7], especially among adolescents [8]. Mobile health and digital medicine have rapidly become an important area of study [9] in response to the conundrum posed by the escalating demand for mental health assistance [10] and the severe shortage of traditional health care facilities [11].

Driven by digital technologies, such as computers, the internet, mobile devices, mobile software apps, and virtual reality (VR), treatment for mental health problems has undergone an unprecedented transformation [12].

A chatbot, as a novel digital technology for mental health service, is a software program that simulates conversations with users through text or voice depending on artificial intelligence (AI) [13]. The first chatbot, ELIZA, was applied in the field of psychology, and users could input text to simulate a conversation with a Rogerian psychotherapist [14]. A mental health chatbot provides more accessibility than traditional face-to-face counseling and psychotherapy [15,16], through which users can feel accompanied and understood [17,18]. In addition, chatbots were designed to focus on interactive capabilities instead of single psychological education for facilitating the process of psychotherapy [19]. Most mental health chatbots can independently provide service to users, without requiring the participation and guidance of human therapists [20]. However, studies have shown that mental health chatbots have some risks as well, such as “misunderstanding,” which may lead to ineffective or even harmful interventions, lack of crisis warning mechanisms, and lack of privacy protection [21]. Chatbots in mental health are nascent [22], and although chatbots have demonstrated feasibility to provide mental health treatment, more high-quality evidence regarding the effectiveness and acceptability of mental health chatbots is needed [23], particularly during the COVID-19 pandemic [24].

According to the latest data of the World Health Organization, there were 3.22 million depressed people worldwide in 2015 [25]. In China, the figure has been reported to be 95 million [26], and the prevalence among college students was 28.4% [27], reaching 34% during the COVID-19 pandemic [28]. However, the use of health services for depressive disorders in China has been rather limited, with the access rate of adequate treatment being less than 0.5% [29]. Cognitive behavioral therapy (CBT) has been continuously developed and is currently recognized as a widespread and effective evidence-based psychotherapy for depression [30,31], serving as one of the crucial theoretical frameworks for chatbot interventions. In recent years, a number of mental health chatbots have emerged, and their effectiveness has been tested through randomized controlled trials, providing interventions for different mental health problems, with Woebot [32], Tess [33,34], Wysa [35], Vivibot [36], and XiaoNan [37] directly targeting depression and anxiety symptoms; Shim [38], SABORI [39], and Bella [40] directly targeting stress, well-being, or quality of life; and MYLO [41-43] and Help4Mood [44] directly targeting general psychological distress such as problem solving and negative cognition.

The technology and format of chatbots for mental health problems have evolved from script bots with only text communication to embodied conversational agents [45] with image and voice, and digital humans [40] and virtual humans [46], which discern and control emotional and facial expressions during interactions with individuals in real time, have also been reported.

However, previous studies focused more on the role of a chatbot as a technical carrier in the intervention, neglecting the verification and innovation of the psychological process and content itself. As a result, there is a gap between the progress of psychology and AI in the field of digital mental health. Chatbots are intended to foster collaboration, integration, and co-development between psychological science and other fields [47]. Thus, a direct comparison between mental health chatbots and general chatbots is essential in a trial. Methodological limitations that existed in previous trials involved an insufficient sample size, a lack of follow-up assessment, failure to comprehensively investigate the long-term effectiveness of the intervention, and ignorance of the sensitivity analysis to ensure robustness of the conclusion.

As an alternative and useful precursor to clinical effectiveness, nonclinical metrics are just as important as clinical outcomes and may contribute to further exploring the mechanism by which the mental health chatbots work [48]. Fitzpatrick et al [32] also noted that therapeutic process factors of mental health chatbots may facilitate or undermine the treatment. From the technical perspective, there is currently no standard method in use to evaluate mental health chatbots. As a result, we attempted to establish a systematic evaluation of nonclinical metrics for mental health chatbots covering adherence, engagement, working alliance, usability, acceptability, and thematic analysis of users’ feedback. Working alliance (also known as “therapeutic alliance”) represents the cooperative and emotionally connected relationship between the client and the therapist, and is considered a common factor in psychotherapy outcomes [49] and a metric to assess the computer-patient relationship as well [50,51]. Three recent studies [52-55] by Dosovitsky et al, Beatty et al, and Darcy et al had emphasized the viability and significance of the relationship and working alliance in digital treatment, and several randomized controlled trials of mental health chatbots had employed the Working Alliance Inventory (WAI) as a measurement method of working alliance, with all of these demonstrating good measure effects [37,56,57]. Important issues to be addressed for chatbots in the future could be extracted from the perceptions and opinions of patients [58], and thematic analysis with a topic model is a qualitative research method to accurately capture and concisely present key information in texts [59].

A randomized controlled trial including 148 Chinese college students was conducted in this study to evaluate the performance and efficacy of a mental health chatbot (XiaoE) for depression. We expected that, compared with an e-book and a general chatbot, the mental health chatbot would be more effective in reducing depressive symptoms after 1-week treatment and that this effect would persist for 1 month after the intervention (primary hypothesis). Additionally, we hypothesized that the mental health chatbot would make it easier to build relationships with users, enhance engagement, and improve user experience during the therapeutic process (secondary hypothesis).

## Methods

### Study Design and Participants

The study was a single-blind, 3-arm randomized controlled trial performed at a university in Tianjin, China. College students were recruited from social media outlets, online platforms, and university communities or were referred here by their counselor in the counseling center. All potential participants were screened by counseling psychologists for eligibility against the following inclusion criteria: (1) age 17-34 years; (2) average score of the depression subscale in the College Students Mental Health Screening Scale (CSMHSS) [60] within 2 to 3; and (3) ability to read Chinese. Participants were excluded if they (1) reported a score of ≥3 for any item in the suicide subscale in the CSMHSS; (2) reported a standard score of >3 in the suicide subscale or hallucination/delusion subscale in the CSMHSS; or (3) were taking a psychiatric medication. The CSMHSS is the mainstream tool for mental health screening of college students in China. The screening scale includes 22 dimensions that involve the main mental health problems of college students and is divided into 3 levels of screening that indicate 3 levels of mental health risk. The CSMHSS is a relevant tool for the inclusion criteria because it can not only measure the degree of depressive symptoms but also screen out individuals with high mental health risk for exclusion. Moreover, the CSMHSS is easier to implement in a university with the help of corresponding assessment platforms, given the large number of recruits. Before the enrollment, the participants were required to carefully read and sign the written informed consent form to confirm their acceptance of the study. Participants were provided with access to artificial psychological counseling services if they had any risk of suicide, self-injury, or severe psychological distress during or after the trial, to avoid further damage. At the end of the trial, participants in control conditions were offered access to XiaoE. The trial was prospectively registered with the ChiCTR registry on October 30, 2021 (number: ChiCTR2100052532). Final data were collected on December 16, 2021. Participants received a compensation of RMB 70 (approximately US $10) for their participation in this trial.

### Randomization and Masking

Randomization with stratification by gender was performed via computer programs independently developed by the technical development team of XiaoE. Participants who were randomly assigned (1:1:1) to receive the mental health chatbot intervention, e-book intervention, or general chatbot intervention would automatically enter the corresponding intervention process when they checked into XiaoE for the first time. Treatment allocation was masked from participants, investigators, and those involved in analyzing trial data, as it was saved in an encrypted electronic file form by multiple parties (the study designer, trial implementer, data processor, and technical development representative) and unblinded after the completion of data analysis. The intervention as well as the outcome measure were completed online, and none of the investigators had access to the participants’ systems during the intervention period (single blind).

### Procedure

The intervention lasted for 1 week. On the day of enrollment (T0), baseline data were collected, including a pretest of the primary outcome measure (9-item Patient Health Questionnaire [PHQ-9]) and demographic information. A posttest of the primary outcome was performed 1 week later (T1), accompanied by the secondary outcomes working alliance, usability, and acceptability. A final follow-up assessment of the primary outcome was carried out 30 days after enrollment (T2).

#### XiaoE

XiaoE is an unguided CBT-based chatbot developed for depression, which can be used in screening, prevention, and self-assistance for depressive symptoms through a fully automatic intelligent interaction with users (text, image, and voice). The technology of XiaoE is rooted in natural language processing (NLP) and deep learning [61]. The whole chatbot dialogue system has been constructed through the open-source framework RASA [62], with content about mental health produced, discussed, and supervised by a psychologist panel led by several experienced clinical and counseling experts from schools and hospitals. XiaoE provides self-assistance service via the WeChat Official Accounts Platform. The objective of the development of XiaoE is not to replace human therapists, but to provide a convenient self-help intervention to users failing to receive immediate mental health services. It can also serve as an auxiliary tool to cooperate with traditional psychological counseling and treatment, covering functions including campus and epidemic-related counseling, adolescent mental health screening and diagnostics, automated CBT-based chatbot interventions, intelligent multiturn conversations, artificial psychological counseling, and “tree hole” (a place to share thoughts and secrets). Participants in this condition were exposed to only the automated CBT-based chatbot intervention. Based on the principles of CBT, multiturn dialogue [63] and personalized customization were taken as the main intervention forms by referring to the content and process of several mature CBT-based chatbots [20] and internet-delivered cognitive behavioral therapy (ICBT) apps [64]. The following 7 modules were designed: “Cognition Challenge,” “Improve Self-esteem,” “Learn to Relax,” “Energy List,” “Wonderful World,” “Are You OK,” and “Escape from Loneliness,” and they correspond to the 7 concepts of psychology, cognitive distortions, self-esteem, mindfulness meditation, mental energy, natural connection, self-help, and loneliness, respectively. Participants were asked to complete a module per day in sequence during the 1-week intervention period, as well as a separate module called “Gratitude Journal” for recording positive events and mood every day.

XiaoE is equipped with complete process guidance and daily task reminders. During the implementation of the trial, the participants were only required to follow the guidance of XiaoE every day, where the staff only provided answers to technical or operational questions. In addition, the interaction data of engagement and use of XiaoE can be obtained in the background of the system. The data could not be obtained from the control groups because the interactions occurred outside the XiaoE system. As a result, the interaction frequency in the control groups was measured in the form of a self-rating questionnaire at the end of the trial.

#### e-Book

Participants in control group 1 were arranged to read an e-book about depression, I Had a Black Dog [65], which is a classic book that introduces depression knowledge to the public and guides to help depressed patients serve themselves from the first-person perspective of depressed patients and their companions. The World Health Organization adopted the animated version as its official promotional video [66] on the theme of depression. In addition, participants in the group were presented with a high-quality depression-related article daily, with the theme of each article corresponding to the daily theme of the functional modules of the intervention group.

#### Xiaoai

Participants in control group 2 were asked to communicate with Xiaoai at least once a day. Xiaoai is a chatbot in China designed to cater to the demands of a wider audience for small talk and not particularly for mental health services such as depression. The chat content between participants and Xiaoai was unrestricted. However, we limited the daily conversation topics (corresponding to the daily functional modules of the intervention group) and proposed specific chat tasks to the participants. For example, the topic on day 2 was self-esteem, and we endorsed that participants share their perspectives and feelings on self-esteem with Xiaoai, discuss “how self-esteem affects our emotional state and what is the relationship between it and depression,” assess their current level of self-esteem with Xiaoai, and ask for advice on “how to improve it.”

### Outcomes

#### Primary Outcome

The primary outcome was the score of the PHQ-9 [67], which is one of the most widely used, reliable, and validated measures of depressive symptoms. It is a 9-item self-report questionnaire that assesses the frequency and severity of depressive symptoms within the previous 2 weeks based on the Diagnostic and Statistical Manual of Mental Disorders, 4th edition (DSM-IV) criteria for major depressive disorder on a 4-point scale from 0 (not at all) to 3 (nearly every day). Scores ranging from 0 to 5 indicate no symptoms of depression, and scores of 5-9, 10-14, 15-20, and 20 represent mild, moderate, moderately severe, and severe depression, respectively.

#### Secondary Outcomes

The secondary outcomes were the scores of the Working Alliance Questionnaire (WAQ) [68], the Usability Metric for User Experience-LITE (UMUX-LITE) [69], and the Acceptability Scale (AS). The WAQ is based on the Helping Alliance Questionnaire (HAq-II), WAI, and California Psychotherapy Alliance Scales (CAL-PAS), with three 4-item subscales assessing the development of an affective bond in treatment and the level of agreement with treatment goals and treatment tasks. The scores of all 12 items range from 0 (rarely) to 5 (always). Usability, as “the extent to which a product can be used by specified users to achieve specific goals with effectiveness, efficiency, and satisfaction in a specified context of use” [70], was assessed by the UMUX-LITE, with 2 items to assess usefulness and ease of use, respectively, ranging from 0 (rarely) to 5 (always). Acceptability, referring to psychological acceptability for the therapeutic process and content, was assessed using a 5-point Likert scale (AS), referring to items used in previous studies on mental health chatbots [33,71] covering overall satisfaction, content satisfaction, emotional awareness, learning new knowledge, relevance to daily life, and promotion of the self-help process.

### Statistical Analyses

Sample size calculation was conducted with G* Power (version 3) [72]. Latest research showed a large effect (*d*=0.83) of a chatbot intervention for depression in college students [37]. On the assumption that a replication study might be expected to achieve broadly similar results, we calculated that a sample size of 32 in each group would have 90% power to detect a net effect size of 0.83, using analysis of covariance (ANCOVA) with a 2-sided significance level of .05, while also allowing for a 20% loss to follow-up.

Difference tests were conducted with SPSS (version 26; IBM Corp). In order to determine whether any significant differences between groups existed at baseline, *F* tests with one-way analysis of variance (ANOVA) were performed on continuous baseline variables (PHQ-9 and age), and chi-square analyses were performed on categorical or nominal variables (gender, ethnicity, only child, single parent, religion, home location, and parental marriage). The same comparisons of baseline characteristics were conducted between dropouts and participants who completed the study. Adjusted mean changes in the PHQ-9 score from baseline to T1 and T2 were analyzed as the primary efficacy endpoint using an ANCOVA model with the treatment group as the fixed effect and the corresponding baseline value as the covariate. A covariate was removed from the statistical model in case of significant interaction effects being found between this covariate and the group. A post-hoc test with Bonferroni correction was employed for multiple group comparisons. η^2^ was calculated and converted to Cohen *d* to examine the effect size of the group difference [73]. A Cohen *d* of 0.2 represents a small effect; 0.5, a moderate effect; and 0.8, a large effect [74]. *F* tests with ANOVA were performed for the results of secondary outcomes.

The results of both the intention-to-treat (ITT) analysis [75] on the full analysis set (all enrolled participants) and the per-protocol (PP) analysis on the PP set (participants in the full analysis set without important protocol violations leading to exclusion) were reported by including all available observations in the analysis [76]. Using *mi impute* within Stata (version 15; StataCorp), we processed missing data via multiple imputation (MI) methods and performed further sensitivity analysis via δ-based methods [77].

There are 3 broad classes of missing data mechanism assumptions [78]: missing completely at random (MCAR), missing at random (MAR), and missing not at random (MNAR). MI is based on MAR, where the probability of a datum being missing does not depend on the unobserved value of the datum, but only depends on the observed values of other recorded variables. Nevertheless, missing data may not necessarily conform to MAR. Instead, they may follow MNAR, where the probability of a datum being missing does depend on the unobserved value of the datum, even given the observed data. We cannot distinguish between MNAR and both MAR and MCAR since the true values of missing data are never known, which means the results of MI may be biased. The publication of ICH E9 (R1) [79], addendum on estimands and sensitivity analysis in clinical trials, states that sensitivity analysis of missing data should be performed to ensure the robustness of the results. As a result, we performed a sensitivity analysis with δ-based methods to see if the effect remained significant when missing data followed MNAR. δ-based MI entails modifying the MAR imputation distribution using a specified numerical delta parameter to make predicted responses better or worse than predicted under MAR. For a continuous outcome, δ, the offset parameter can represent the difference in the mean response between the observed and unobserved cases [80]. Usually, the sensitivity analysis will repeat for a range of δ values corresponding to 25%, 50%, 75%, and 100% of the absolute change from baseline of outcomes in all participants.

Adherence is revealed by chi-square analysis of the attrition of participants, and engagement is revealed by the frequency and duration of the interaction with the chatbot. An interaction was considered a session if there was engagement with the chatbot lasting at least 2 user inputs within 2 minutes and a break no longer than 1 minute. Mean interaction frequency was defined as the average number of sessions each participant had with the chatbot per day during the 1-week intervention period. Mean interaction duration was defined as the average response time of each session calculated in milliseconds between the first time the user inputs content and the last time the chatbot outputs content per day. The 1-week intervention period was divided into days 1 through 7, and each day’s 24 hours were divided into 12 two-hour time periods. We recorded and calculated the mean interaction frequency and mean interaction duration for the 7 days and the 12 time periods. We recontacted all the enrolled participants after all the follow-up measurements were finished and opened access to XiaoE. They were asked 3 open-ended questions at the end of the trial: “What was your best experience using XiaoE?” “What was your worst experience using XiaoE?” and “Please make some personal comments or suggestions on XiaoE.” We ran a thematic analysis on participants’ feedback using Latent Dirichlet Allocation (LDA) [81], an unsupervised learning algorithm, with Pycharm (version 2020.2.2). In order to confirm the optimal number of themes for participants’ feedback on each question, the perplexity under different numbers of themes should be calculated, and the topic model with the minimum perplexity should be selected. Five keywords were extracted from each theme, and each theme was named by combining keywords and original feedback text labeled as corresponding themes.

### Ethics Approval

The study protocol was approved by the Medical Ethics Committee of Tianjin Anding Hospital (Tianjin Mental Health Center; number: 2021-21). All participants provided informed consent.

## Results

### Participant Characteristics

**Figure 1:**
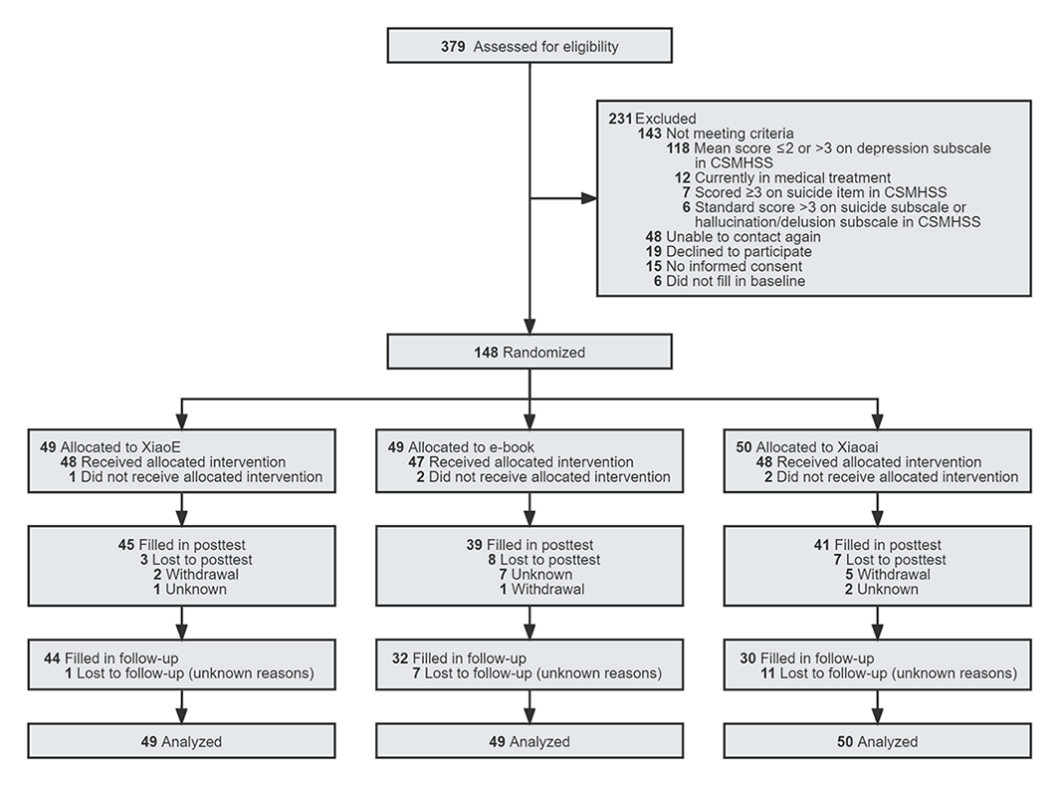
Flow of participants (CONSORT). CSMHSS, College Students Mental Health Screening Scale.

Figure 1 shows the participant flow (CONSORT flow diagram) [82]. A total of 379 college students were assessed for eligibility and enrolled between September 1, 2021, and November 15, 2021, of whom 143 did not meet the study criteria, 48 could not be contacted again, 19 declined to participate, 15 did not sign the written informed consent form, and 6 failed to complete the baseline measure. Ultimately, 148 participants were enrolled and randomized, of whom 49 were allocated to use the mental health chatbot (XiaoE), 49 were allocated to read the e-book, and 50 were allocated to use the general chatbot (Xiaoai). Participants were on average 18.78 years old (SD 0.89; range 17-21 years), and 37.2% (55/148) were female. The mean PHQ-9 score was 10.02 (SD 3.18; range 2-19) at baseline, just reaching the level of moderate depression. There were no significant differences in baseline characteristics among the 3 arms, as well as between dropouts and participants who completed the study (Table 1). Five participants (1 from the XiaoE group and 4 from the Xiaoai group) were identified by counseling psychologists as high risk during and after the course of the trial and underwent artificial psychological counseling.

**Table 1:** Baseline characteristics by randomization arm.

Characteristic			XiaoE (N=49)		e-book (N=49)		Xiaoai (N=50)		Total (N=148)		*F*/χ^2^ (df)^b^		*P* value
PHQ-9^a^ score, mean (SD)			10.10 (3.18)		9.18 (3.94)		10.76 (3.86)		10.02 (3.71)		2.294 (2,145)		.11
Age, mean (SD)			18.80 (0.89)		18.92 (0.84)		18.64 (0.90)		18.78 (0.88)		1.258 (2,145)		.29
**Gender, n (%)**											0.023 (2)		.99
	Male	31 (63.3)		31 (63.3)		31 (62.0)		93 (62.8)					
	Female	18 (36.7)		18 (36.7)		19 (38.0)		55 (37.2)					
**Ethnicity, n (%)**											3.239 (2)		.20
	Han	44 (89.8)		44 (89.8)		49 (98.0)		137 (92.6)					
	Non-Han	5 (10.2)		5 (10.2)		1 (2.0)		11 (7.4)					
**Only child, n (%)**											2.043 (2)		.36
	Yes	16 (32.7)		13 (26.5)		10 (20.0)		39 (26.3)					
	No	33 (67.3)		36 (73.5)		40 (80.0)		109 (73.7)					
**Single parent, n (%)**											0.450 (2)		.80
	Yes	6 (12.2)		4 (8.2)		5 (10.0)		15 (10.1)					
	No	43 (87.8)		45 (91.8)		45 (90.0)		133 (89.9)					
**Religion, n (%)**											1.912 (2)		.38
	Yes	3 (6.1)		4 (8.2)		1 (2.0)		8 (5.4)					
	No	46 (93.9)		45 (91.8)		49 (98.0)		140 (94.6)					
**Home location** **, n (%)**											5.057 (4)		.28
	Urban	11 (22.5)		14 (28.6)		12 (24.0)		37 (25.0)					
	Suburban	10 (20.4)		6 (12.2)		15 (30.0)		31 (20.9)					
	Rural	28 (57.1)		29 (59.2)		23 (46.0)		80 (54.1)					
**Parental marriage, n (%)**											6.089 (4)		.19
	Harmony	36 (73.5)		42 (85.7)		45 (90.0)		123 (83.1)					
	Disharmony	7 (14.3)		5 (10.2)		2 (4.0)		14 (9.5)					
	Divorced	6 (14.2)		2 (4.1)		3 (6.0)		11 (7.4)					

^a^PHQ-9: 9-item Patient Health Questionnaire.

^b^*F* value for PHQ-9 and age, and ^2^ for gender, ethnicity, only child, single parent, religion, home location, and parental marriage.

### Adherence and Attrition

Of the 49 participants allocated to the XiaoE group, 4 dropped out over the 1-week period and 1 dropped out over the 1-month period. Of the 49 participants allocated to the e-book group, 10 dropped out over the 1-week period and 7 dropped out over the 1-month period. Of the 50 participants allocated to the Xiaoai group, 9 dropped out over the 1-week period and 11 dropped out over the 1-month period (Figure 1). There was a lower attrition in the intervention condition compared with the control conditions (37% vs 10%; ^2^_1_=11.904; *P*<.001).

### Effectiveness

#### ITT Analysis

At T1, no significant interaction effects were found between group and baseline PHQ-9 score (*P*=.86), age (*P*=.91), gender (*P*=.32), ethnicity (*P*=.20), only child (*P*=.33), single parent (*P*=.99), religion (*P*=.54), home location (*P*=.62), and parental marriage (*P*=.59) with the ANCOVA model. Similarly, at T2, no significant interaction effects were found between group and baseline PHQ-9 score (*P*=.16), age (*P*=.14), gender (*P*=.43), ethnicity (*P*=.96), only child (*P*=.27), single parent (*P*=.59), religion (*P*=.87), home location (*P*=.90), and parental marriage (*P*=.66) with the ANCOVA model.

**Figure 2:**
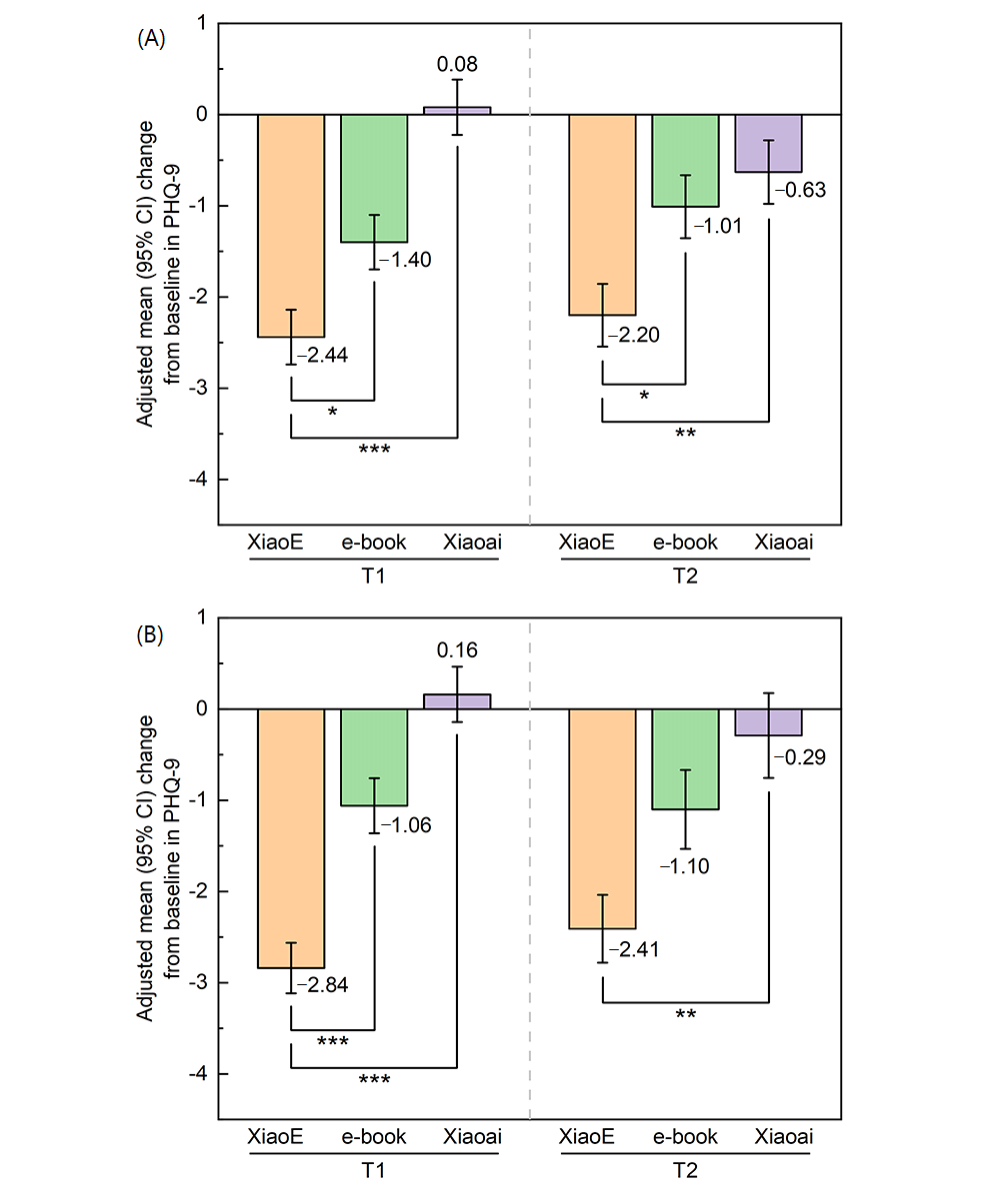
Efficacy for the reduction of depression symptoms in participants. The image presents the mean change from baseline in the primary outcome measure (9-item Patient Health Questionnaire [PHQ-9]) and the between-group differences in participants with XiaoE versus those with e-book and Xiaoai at postintervention and at follow-up. Means and standard errors are displayed. (A) Intention-to-treat analysis. (B) Per-protocol analysis. *P<.05; **P<.01; ***P<.001.

Depressive symptoms significantly reduced more among participants in the XiaoE group in comparison with controls, and a moderate between-group effect size was reported at T1 (*F*_2,136_=17.011; *P*<.001; *d*=0.51), while a small effect size was reported at T2 (*F*_2,136_=5.477; *P*=.005; *d*=0.31) (Table 2). The post-hoc test with Bonferroni correction revealed significant treatment differences with XiaoE versus e-book and Xiaoai in the reduction of depression at T1 (*P*=.04 and *P*<.001, respectively) and T2 (*P*=.049 and *P*=.006, respectively) (Figure 2).

All results were robust under sensitivity analysis, except for the comparison with e-book at T2, which changed from significant to not significant (Table 3).

**Table 2:** Primary outcome measures and between-group differences in the full analysis set and per-protocol set.

Analysis and timepoint					XiaoE							e-book							Xiaoai							*F* (df)				*P* value				η^2^				Cohen’s *d*
					Adjusted^a^ PHQ-9^b^, mean (SE)			n			Adjusted^a^ PHQ-9^b^, mean (SE)				n			Adjusted^a^ PHQ-9^b^, mean (SE)				n																
**ITT^c^ analysis**																																						
	**Postintervention**			7.58 (0.30)			45			8.62 (0.30)				39			10.10 (0.30)				41			17.011 (2,136)				<.001				0.060				0.51		
		Change from baseline	−2.44 (0.30)						−1.40 (0.30)							0.08 (0.30)																						
	**Follow-up**			7.82 (0.34)			44			9.01 (0.35)				32			9.39 (0.35)				30			5.477 (2,136)				.005				0.024				0.31		
		Change from baseline	−2.20 (0.34)						−1.01 (0.35)							−0.63 (0.35)																						
**PP^d^ analysis**																																						
	**Postintervention**			7.51 (0.28)			45			9.29 (0.30)				39			10.51 (0.30)				41			26.168 (2,113)				<.001				0.088				0.62		
		Change from baseline	−2.84 (0.28)						−1.06 (0.30)							0.16 (0.30)																						
	**Follow-up**			7.92 (0.37)			44			9.23 (0.43)				32			10.04 (0.46)				30			6.408 (2,94)				.002				0.044				0.43		
		Change from baseline	−2.41 (0.37)						−1.10 (0.43)							−0.29 (0.46)																						

^a^Adjusted for baseline PHQ-9 score, age, gender, ethnicity, only child, single parent, religion, home location, and parental marriage.

^b^PHQ-9: 9-item Patient Health Questionnaire.

^c^ITT: intention-to-treat.

^d^PP: per-protocol.

**Table 3:** δ-based sensitivity analysis.

Time and analysis^a^		Compared to e-book				Compared to Xiaoai			
		Group difference, value (SE)	95% CI	*P* value	Group difference, value (SE)		95% CI	*P* value	
**T1 (after 1 week)**									
	MI^b^, MAR^c^	−1.52 (0.43)	−2.38 to −0.66	.001	−2.62 (0.42)		−3.45 to −1.78	<.001	
	δ=−0.31	−1.45 (0.43)	−2.31 to −0.59	.001	−2.52 (0.42)		−3.36 to −1.69	<.001	
	δ=−0.62	−1.38 (0.44)	−2.25 to −0.51	.002	−2.43 (0.43)		−3.27 to −1.58	<.001	
	δ=−0.93	−1.31 (0.44)	−2.19 to −0.43	.004	−2.33 (0.43)		−3.18 to −1.48	<.001	
	δ=−1.24	−1.24 (0.45)	−2.13 to −0.35	.007	−2.24 (0.44)		−3.10 to −1.37	<.001	
**T2 (after 1 month)**									
	MI, MAR	−1.11 (0.54)	−2.18 to −0.03	.043	−1.65 (0.55)		−2.74 to −0.56	.003	
	δ=−0.32	−1.03 (0.54)	−2.11 to 0.04	.06	−1.55 (0.55)		−2.64 to −0.46	.006	
	δ=−0.64	−0.96 (0.55)	−2.05 to 0.12	.08	−1.45 (0.55)		−2.55 to −0.35	.01	
	δ=−0.96	−0.89 (0.55)	−1.98 to 0.20	.11	−1.35 (0.56)		−2.46 to −0.24	.02	
	δ=−1.28	−0.81 (0.56)	−1.92 to 0.29	.15	−1.25 (0.56)		−2.37 to −0.13	.03	

^a^The absolute mean change from baseline to postintervention in the PHQ-9 score of all participants was −1.24, and the absolute mean change from baseline to follow-up in the PHQ-9 score of all participants was −1.28.

^b^MI: multiple imputation.

^c^MAR: missing at random.

#### PP Analysis

At T1, no significant interaction effects existed between group and baseline PHQ-9 score (*P*=.59), age (*P*=.88), gender (*P*=.47), ethnicity (*P*=.44), only child (*P*=.39), single parent (*P*=.86), religion (*P*=.69), home location (*P*=.21), and parental marriage (*P*=.57) with the ANCOVA model. Similarly, at T2, no significant interaction effects existed between group and baseline PHQ-9 score (*P*=.34), age (*P*=.30), gender (*P*=.98), ethnicity (*P*=.95), only child (*P*=.11), single parent (*P*=.37), religion (*P*=.68), home location (*P*=.53), and parental marriage (*P*=.52) with the ANCOVA model.

Depressive symptoms significantly reduced more among participants in the XiaoE group in comparison with controls, and a moderate between-group effect size was reported at T1 (*F*_2,113_=26.168; *P*<.001; *d*=0.62), while a small effect size was reported at T2 (*F*_2,94_=6.408; *P*=.002; *d*=0.43) (Table 2). The post-hoc test revealed significant treatment differences with XiaoE versus e-book and Xiaoai in the reduction of depression at T1 (*P*<.001 and *P*<.001, respectively) and a significant difference between XiaoE and Xiaoai (*P*=.003) but no significant difference between XiaoE and e-book (*P*=.08) at T2 (Figure 2).

### Use and Engagement

**Figure 3:**
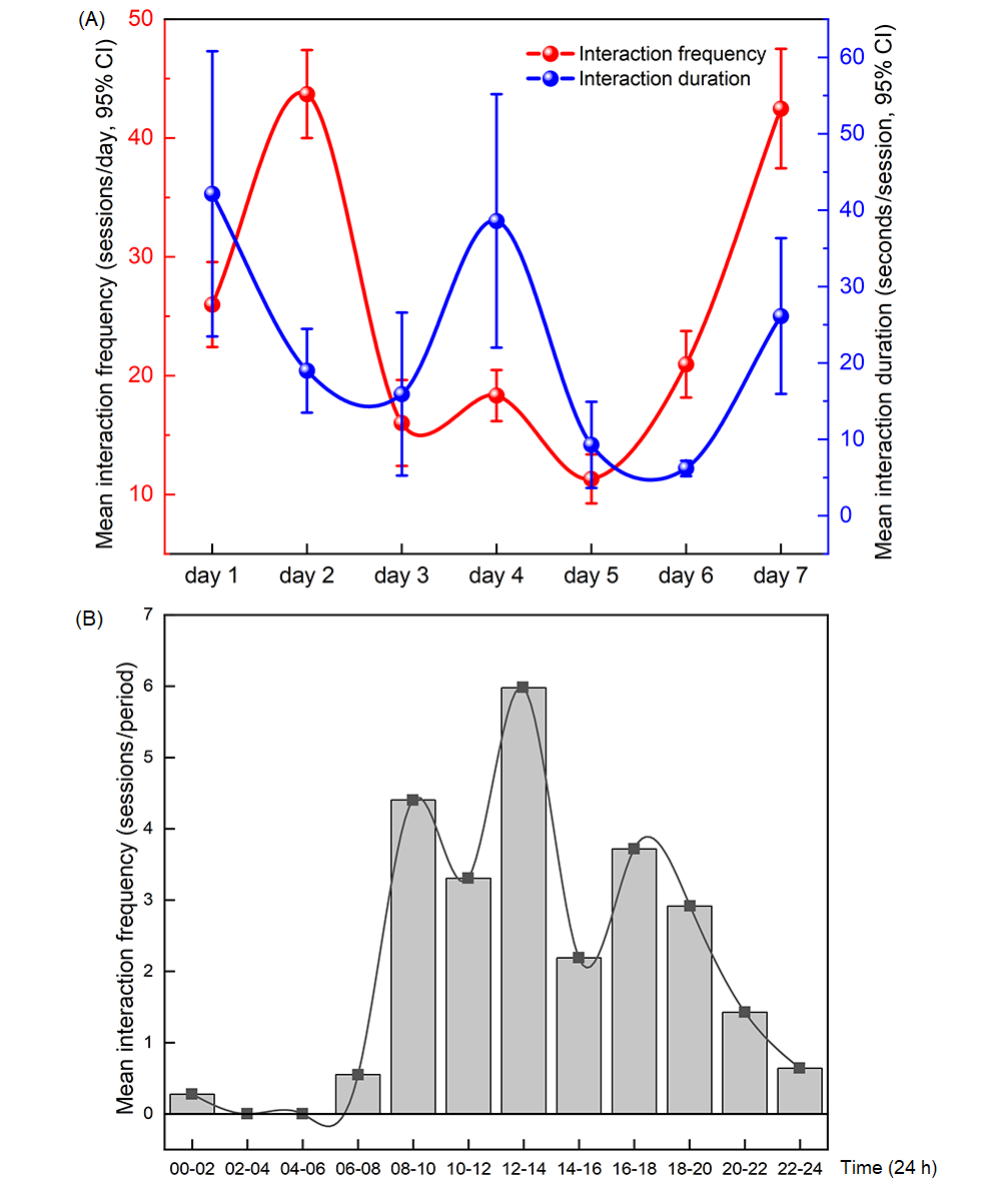
Use and engagement with XiaoE. The image shows the frequency and duration of interaction with the chatbot and the trend of daily interactions and interactions for 12 time periods per day in the XiaoE group during the intervention. (A) Daily engagement. The x-axis represents each day of the 1-week intervention. (B) Engagement for 12 time periods. The x-axis represents each time period in 1 day.

As shown in Figure 3, participants in the XiaoE group interacted with the chatbot for 25.54 sessions (SD 26.45; range 0-172) on average per day, and each session lasted an average of 22.46 seconds (SD 79.88; range 0-758 seconds) over the 1-week period. The daily frequency and duration of the interaction were high on day 1, day 2, and day 7, while they were relatively low on day 3, day 5, and day 6, and rebounded to some extent on day 4. The frequency of the interaction reached peaks in the 3 time periods of 8-10 AM, 12-2 PM, and 4-6 PM per day. According to the answers of participants in the e-book group, 2% (1/49) had not read it once, 51% (25/49) had read it once, and 47% (23/49) had read it twice or more. In the Xiaoai group, 29% (14/48) said they interacted with Xiaoai once a day, 27% (13/48) said twice a day, and 44% (21/48) said 3 or more times a day.

### Working Alliance, Usability, and Acceptability

Table 4 summarizes the results of the secondary outcomes. Participants in the XiaoE condition scored higher on the total WAQ (*F*_2,145_=3.407; *P*=.04), as well as the subscales Bond (*F*_2,145_=3.890; *P*=.02) and Engagement (*F*_2,145_=3.925; *P*=.02) compared with the e-book group and the Xiaoai group. No significant difference among arms was found on the UMUX-LITE (*F*_2,145_=0.968; *P*=.38). Better acceptability was discovered in the XiaoE group for total AS (*F*_2,145_=4.322; *P*=.02), content satisfaction (*F*_2,145_=5.093; *P*=.007), emotional awareness (*F*_2,145_=3.636; *P*=.03), learning new knowledge (*F*_2,145_=4.330; *P*=.02), and relevance to daily life (*F*_2,145_=4.834; *P*=.009).

**Table 4:** Secondary outcome measures and differences between conditions.

Variable		XiaoE (n=49), mean (SD)	e-book (n=49), mean (SD)	Xiaoai (n=50), mean (SD)	*F* (df)	*P* value
**WAQ^a^ score**						
	Total	53.94 (5.96)	50.35 (9.38)	50.68 (6.87)	3.407 (2,145)	.04
	Goal task	17.22 (2.71)	16.43 (3.10)	16.54 (2.48)	1.188 (2,145)	.31
	Bond	18.47 (1.92)	17.06 (3.26)	17.32 (2.64)	3.890 (2,145)	.02
	Engagement	18.24 (2.25)	16.86 (3.54)	16.82 (2.69)	3.925 (2,145)	.02
**UMUX-LITE^b^ score**						
	Total	8.61 (1.43)	8.31 (1.52)	8.24 (1.30)	0.968 (2,145)	.38
	Usefulness	4.16 (0.94)	4.14 (0.76)	4.08 (0.78)	0.135 (2,145)	.87
	Ease of use	4.45 (0.71)	4.16 (0.87)	4.16 (0.77)	2.192 (2,145)	.12
**AS^c^ score**						
	Total	27.86 (3.25)	25.82 (5.04)	25.48 (4.53)	4.322 (2,145)	.02
	Overall satisfaction	4.67 (0.75)	4.43 (0.89)	4.32 (0.89)	2.264 (2,145)	.11
	Content satisfaction	4.76 (0.52)	4.45 (0.79)	4.30 (0.81)	5.093 (2,145)	.007
	Emotional awareness	4.57 (0.74)	4.20 (1.00)	4.12 (0.90)	3.636 (2,145)	.03
	Learning new knowledge	4.63 (0.64)	4.27 (0.95)	4.16 (0.89)	4.330 (2,145)	.02
	Relevance to daily life	4.67 (0.63)	4.14 (1.10)	4.30 (0.81)	4.834 (2,145)	.009
	Promotion of self-help process	4.55 (0.77)	4.33 (0.94)	4.28 (0.83)	1.429 (2,145)	.24

^a^WAQ: Working Alliance Questionnaire.

^b^UMUX-LITE: Usability Metric for User Experience-LITE.

^c^AS: Acceptability Scale.

### Thematic Analysis

**Figure 4:**
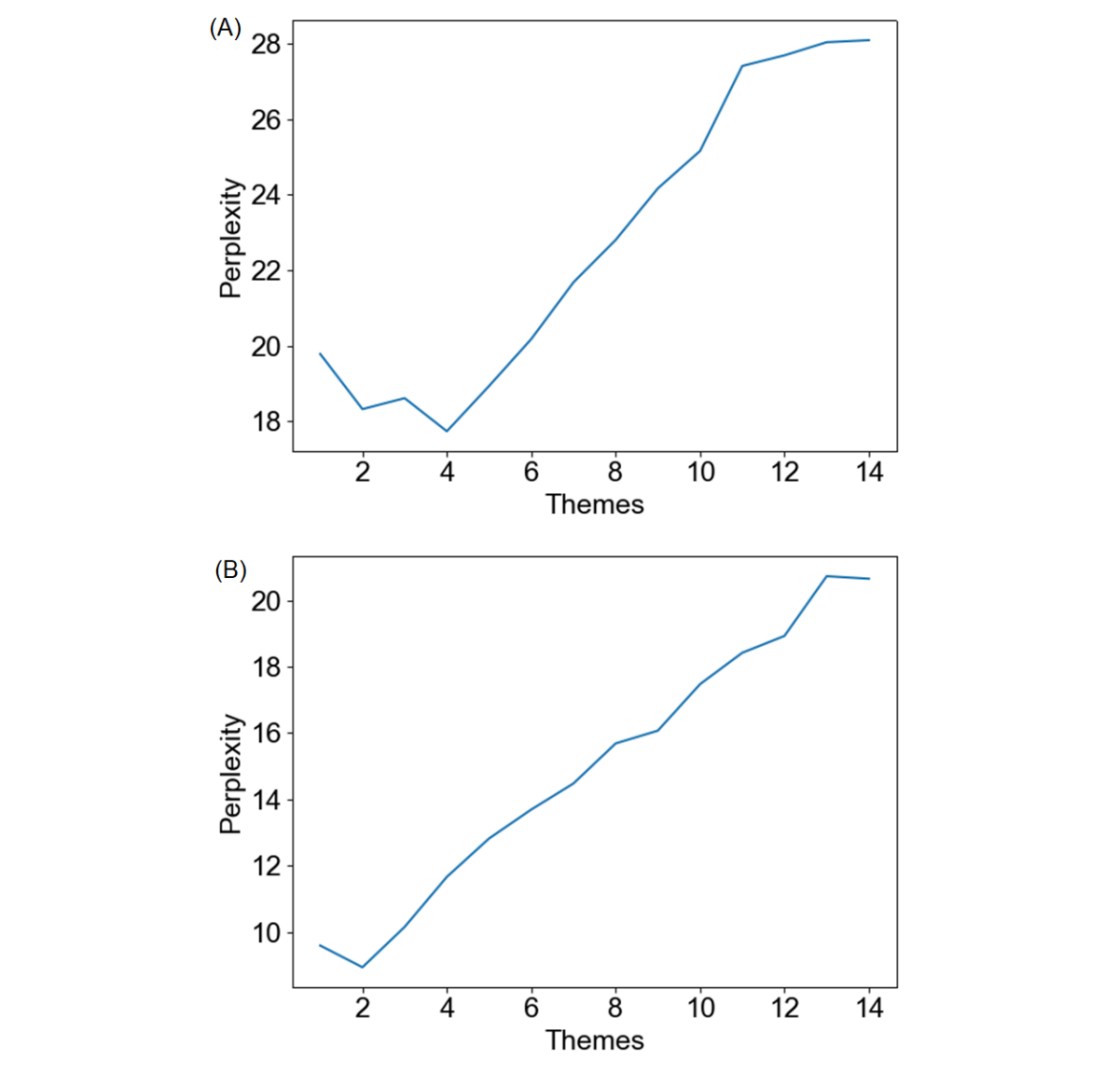
Chart of themes and perplexity. The image shows the perplexity under different number of themes for participant feedback of the 2 questions, "What was your best experience using XiaoE?" and "What was your worst experience using XiaoE?." (A) Themes-perplexity chart of “best experience.” (B) Themes-perplexity chart of “worst experience”.

According to the chart of themes-perplexity of LDA (Figure 4), the number of themes reported in the question “What was your best experience using XiaoE?” was set to 4 and the number of themes reported in the question “What was your worst experience using XiaoE?” was set to 2. Table 5 lists all the themes and keywords for participants’ feedback. The last question “comments or suggestions” was analyzed with a qualitative method because the result of LDA was not ideal.

The following 4 themes emerged in respect to the feedback to the question regarding the *best experience*: “relationship” (n=25), “emotion” (n=12), “personalization” (n=31), and “practicability” (n=80). The keywords extracted from the relationship theme were “company,” “care,” “loneliness,” “favor,” and “attending,” and the corresponding labeled example text was “XiaoE is very sweet, I like to talk to XiaoE, he will accompany and accept me, so I don't feel lonely.” The keywords for the emotion theme were “happy,” “relax,” “stress,” “catharsis,” and “company,” and the example text was “always makes me laugh! Ha ha ha ha, the pressure suddenly disappeared, and I am so happy.” The keywords for the personalization theme were “thinking,” “learning,” “depression,” “mood,” and “intelligence,” and the example was “The best experience is that sometimes XiaoE’s answers are indeed valuable and can really target some of my questions, which is very intelligent and promotes thinking.” The keywords for the practicability theme were “convenience,” “help,” “reality,” “method,” and “usability,” and the example was “practical, real and convenient, can help me.”

The following 2 themes emerged in respect to the feedback to the question regarding the *worst experience*: “content” (n=120) and “technology” (n=28). The keywords extracted from the content theme were “inflexible,” “response,” “tedious,” “repetitive,” and “mechanical,” and the corresponding labeled example text was “The content is too rigid. It will make people feel bored and irritable if used for a long time.” The keywords extracted from the technology theme were “glitches,” “lag,” “system,” “crash,” and “inflexible,” and the example text was “crashed when I just entered the interface, and some glitches need to be optimized.”

The participants’ feedback on the question “comments or suggestions” can be mainly extracted into the following 3 themes: hope for a more fluent process of dialogue, more emotional response and interaction, and server upgrade.

**Table 5:** Themes and keywords for participants’ feedback.

Question and theme			Keywords
**Best experience**			
	Relationship	Company, care, loneliness, favor, and attending	
	Emotion	Happy, relax, stress, catharsis, and company	
	Personalization	Thinking, learning, depression, mood, and intelligence	
	Practicability	Convenience, help, reality, method, and usability	
**Worst experience**			
	Content	Inflexible, response, tedious, repetitive, and mechanical	
	Technology	Glitches, lag, system, crash, and inflexible	

## Discussion

### Principal Findings

To our knowledge, this is the first study to directly compare the clinical efficacy of a mental health chatbot with a general chatbot performing automated teletreatment for depressive symptoms. We tested both the short- and long-term effectiveness of XiaoE via a single-blind, 3-arm randomized controlled trial and established a systematic evaluation of nonclinical metrics for mental health chatbots so as to offer references for future research.

Participants in this trial were on average 18.78 years old, and they were younger than samples of typical studies with adults or college students, indicating that research on mental health chatbots is translating to samples of adolescents. In addition, more men took part in this study than in previous studies, where the majority of participants were women. Given that there are currently no well-done studies on adolescents, we hope to see more of them in the future.

In terms of attrition, participants in the XiaoE group dropped out at a lower rate than those in the e-book and Xiaoai groups. XiaoE was associated with a high level of engagement, which rose to the highest level particularly at the beginning and toward the end of the trial, demonstrating that XiaoE was attractive to participants and could quickly establish relationships when participants came into contact with this novel AI. The participants using XiaoE were most active from 12 to 2 PM every day, which may be related to the automatic task reminder set after 12 PM once a day by XiaoE. However, a large fluctuation in engagement could be observed regarding the trend of weekly activation and daily activation, which indicated that the relationships between participants and XiaoE were not steady and firm enough.

ITT analysis showed a significantly better effectiveness of XiaoE for depression in comparison with that of the 2 controls for 1 week, achieving a moderate effect size (*d*=0.51), which was between the effect sizes of 2 previous studies [32,33] (Woebot: *d*=0.44; Tess: *d*=0.68) and remained robust in sensitivity analysis. The results of the long-term reduction of depressive symptoms 1 month later were statistically significant as well, while achieving a small effect size (*d*=0.31). PP analysis also showed significant short- and long-term effectiveness (T1, *d*=0.62; T2, *d*=0.43). As in previous studies [76], the results of the ITT analysis were lower than those of the PP analysis. However, opposite results were found in separate comparisons of the XiaoE and e-book groups. The difference between XiaoE and e-book was significant in the ITT analysis (despite failing to pass the test of the sensitivity analysis), but not in the PP analysis. Protocol deviations and the interaction between compliance and the intervention, which can lead to better outcomes for compliers in the active group but just the opposite (better for noncompliers) in the control group, are commonly thought to be the causes of the bias in the PP analysis. In this study, nevertheless, the effectiveness for compliers of the e-book group may also be overestimated due to the favorable impact of compliance, which may be more significant than that in the XiaoE group. Therefore, the difference between the 2 groups was not significant in the PP analysis. This shows that mental health chatbots should fortify the therapeutic alliance even more to increase the intervention compliance of participants.

It is necessary to note that while there was a significant improvement in symptoms via the mental health chatbot intervention, the magnitude of the improvement was small. As a result, the mental health chatbot is better suited as an auxiliary tool to work in conjunction with traditional psychological counseling and treatment or as the primary care approach for the treatment of mental illness. Although it is challenging to swiftly implement the intervention in real clinical practice, at least for the time being, the intervention is effective and convenient for individuals who desire to access self-help mental health services. This makes sense, since those represent a much larger group of people, and the spread of this unguided tool will greatly reduce the cost of human and financial resources.

XiaoE exhibited a significant high level of acceptability and work alliance with participants but a nonsignificant high level of usability. This shows that XiaoE has preliminarily reached the standard of capacity in relationship establishment, but some aspects, such as the user interface and the operating system, still need to be further simplified for users. Participants reported having received the best experience with XiaoE in the 4 themes of “relationship,” “emotion,” “personalization,” and “practicality.” The theme of “relationship” reflected the establishment, development, and function of the relationship between XiaoE and participants, as Dosovitsky et al [52] found that individuals can form a positive bond with an AI chatbot owing to its personality traits, such as being caring, open to listening, and nonjudgmental. The theme of “emotion” reflected that communication with XiaoE was helpful for emotional expression and catharsis, and made users feel accompanied and understood. The same themes were also observed in previous studies [32,33]. The theme of “personalization” reflected that XiaoE can make different suggestions for different emotional distresses put forward by the participants, which can trigger more thinking and learning of the participants. At the beginning of the content design of XiaoE, in order to avoid an overly sermonizing feeling, we added many simple and specific tips. This could be the reason why participants considered XiaoE to be practical (“practicality”). The worst experiences reported mainly focused on “content” and “technology.”

As mentioned earlier, the use of psychology in chatbots is still superficial. Despite the fact that our content was based on CBT, we discovered through our thematic analysis that participant comments barely made any mention of it. XiaoE’s conversations do not always emphasize CBT itself to participants, similar to how patients receiving therapy from a human therapist may feel like they are improving but not know what kind of therapy they are actually receiving. On the other hand, it is evident that CBT has its limitations. Even though CBT is a highly structured therapy, translating a typical CBT-based psychotherapy into a chatbot setting is difficult.

### Comparison With Prior Work

We added a general chatbot (Xiaoai) intervention as a control condition to demonstrate the significance of psychological design and content for mental health chatbots. Interestingly, participants who interacted with Xiaoai showed a small worsening of depressive symptoms after receiving the 1-week intervention. This indicates that using a general chatbot to treat mental health problems may be harmful, and a specifically designed chatbot for mental health may be required to alleviate depressive symptoms. In this study, follow-up was added to investigate the long-term effectiveness, and δ-based sensitivity analysis was performed to ensure the robustness of the conclusion. We established an innovative systematic evaluation of nonclinical metrics for mental health chatbots, and LDA was applied for the first time in the thematic analysis of users’ feedback as the sample size increased.

### Limitations and Future Directions

There were some limitations in this study: First, due to the particularity of the tool and the consideration of actual recruitment, it was below capacity to double-blind both the investigators and the participants. For the convenience of management, an online group was set up for the 148 enrolled participants to provide important information and technical solutions during the implementation process of the trial, which, as a potential risk, may have resulted in an attempt to reveal different contents of their own interventions, thus imposing subjective influence on the effectiveness for other participants. Special attention should be paid to this in future online research. Second, the 1-week intervention period in this study was relatively short, and the results might have shown some difference if the intervention was prolonged. It can be concluded from the trend of weekly activation that engagement with XiaoE had a wide fluctuation range and XiaoE showed strong attractiveness, but it rapidly faded in the middle of the trial. It may have resulted from repeated interactions with the inflexible and tedious content, as well as technical problems such as glitches and lag. It can be speculated that the chatbot may be more suitable for a short-term intervention rather than a long-term intervention, which needs to be explored in further studies with a longer treatment period. Third, for the control condition, the strength of evidence for the intervention itself was still limited, and the e-book intervention, as a self-help approach, only involved the concept of psychological education, and it was not equipped with a complete set of programs for psychological therapy [83] or designed for multiple or recurring sessions. Therefore, it is better to choose other active control approaches whose efficacy has been clearly proven, including traditional face-to-face therapy, online psychological counseling, ICBT, and VR. Fourth, as we could only gather self-reported involvement in the control groups as opposed to comprehensive objective data in the treatment group, it was not possible to directly compare the engagement of the XiaoE group with that of the control groups. Future research should also collect behavioral data in control groups corresponding to the data in the treatment group as the basis for comparison. Finally, this study involved students from a single university in Tianjin, China, and it was not determined whether the conclusion can be extended to a larger group. This can be addressed by attempting to perform multicenter randomized controlled trials in the future.

In the postepidemic era, people’s lifestyles have undergone profound transformations, and digital technology and internet informatization have drawn more attention than ever. It is reasonable to predict that in the future, chatbot-based digital psychotherapy will play a significant role in the field of mental health care [84]. This will provide new clinical guidelines and technical viewpoints to relevant psychologists, psychiatrists, and AI researchers and practitioners.

At present, there are many digital therapeutic approaches with excellent psychological content, with little attention to the effective factors in the psychological therapeutic process, such as emotional response, therapeutic alliance, empathy, and personalization. Despite people’s doubt regarding whether machines can provide emotional experiences, they typically respond better to agents that express emotions than those that do not [85], illustrating the importance of a positive therapeutic alliance in the internet environment in the absence of therapist support [86]. Chatbots with sophisticated empathic capabilities can enrich user experience and affinity. The concept of empathic chatbots has been proposed [87], accompanied with system design and development [88], but there is no mature product present and effectiveness has not been tested yet. The utilization of user profiles or user models to support personalized and adaptive features, and assessments for personalization are still limited in mental health chatbots [89]. Thus, the technologies of chatbots, particularly NLP [61] and multiturn dialogue [60], require to be constantly upgraded, and the user interface and operating system should be modified to improve user experience. Future chatbots can be targeted at more mental health problems, such as anxiety, insomnia, well-being, stress, and addiction. Meanwhile, ethical issues with AI, such as privacy, security, information disclosure, and harm avoidance need to be carefully considered [90].

### Conclusions

The mental health chatbot XiaoE can be used as a feasible, engaging, and effective digital intervention for college students with depressive symptoms. Compared with a general chatbot, XiaoE exhibited significant short-term and long-term effectiveness that remained robust after sensitivity analysis, illustrating the unique role of psychological design and process in the field of digital mental health. XiaoE showed special capacity for building relationships with users, enhancing engagement, and improving user experience during the therapeutic process. Further evidence is required to confirm the long-term effectiveness via trails replicated with a longer dose, as well as exploration of its greater efficacy in comparison with other active controls.

## Appendix

Multimedia Appendix 1 CONSORT-eHEALTH checklist (V 1.6.1).

## Abbreviations

AI: artificial intelligence
ANCOVA: analysis of covariance
AS: Acceptability Scale
CBT: cognitive behavioral therapy
CSMHSS: College Students Mental Health Screening Scale
ICBT: internet-delivered cognitive behavioral therapy
ITT: intention-to-treat
LDA: Latent Dirichlet Allocation
MAR: missing at random
MCAR: missing completely at random
MI: multiple imputation
MNAR: missing not at random
NLP: natural language processing
PHQ-9: 9-item Patient Health Questionnaire
PP: per-protocol
UMUX-LITE: Usability Metric for User Experience-LITE
VR: virtual reality
WAQ: Working Alliance Questionnaire
